# Relationship between geriatric nutritional risk index and osteoporosis in type 2 diabetes in Northern China

**DOI:** 10.1186/s12902-022-01215-z

**Published:** 2022-12-09

**Authors:** Yuanyuan JI, Nan Geng, Yingchun Niu, Hang Zhao, Wenjie Fei, Shuchun Chen, Lu ping Ren

**Affiliations:** Department of Endocrinology, Hebei General Hospital, Shijiazhuang, Hebei China

**Keywords:** Geriatric nutrition risk index, Osteoporosis, BMD, Type 2 diabetes mellitus

## Abstract

**Background:**

Osteoporosis is a very common bone disease in the elderly population and can lead to fractures and disability. Malnutrition can lead to osteoporosis. The geriatric nutritional risk index (GNRI) is a tool used to assess the risk of malnutrition and complications associated with nutritional status in older patients and is a crucial predictor of many diseases. Hence, this study investigated the association between the GNRI and the presence of osteoporosis and assessed the value of this index for predicting osteoporosis in patients with type 2 diabetes mellitus (T2DM).

**Methods:**

This cross-sectional study enrolled 610 elderly patients with T2DM. General and laboratory data of the patients were collected, along with their measurements of bone mineral density (BMD). The GNRI was calculated based on ideal body weight and serum albumin (ABL) levels. Correlation analysis was performed to determine the relationship between the GNRI and BMD and bone metabolism indices. The GNRI predictive value for osteoporosis development was analyzed through logistic regression analysis and by creating a receiver operating characteristic curve (ROC), calculating the area under the curve (AUC).

**Results:**

All patients were divided into the no-nutritional risk and nutritional risk groups. Compared with the no-nutritional risk group, the nutritional risk group had a longer diabetes course, older age, higher HbA1c levels, higher prevalence of osteoporosis; lower BMI, ABL,triglyceride (TG),Calcium (Ca),25-hydroxy-vitamin-D(25(OH)D),and parathyroid hormone(PTH) and lower femoral neck BMD,total hip BMD (*P* < 0.05).

All patients were also assigned to the non-osteoporosis and osteoporosis groups. The non-osteoporosis group had higher GNRI values than the osteoporosis group (*P* < 0.05).

Correlation analysis revealed a positive correlation between the GNRI and lumbar BMD, femoral neck BMD, and total hip BMD (*P* < 0.05). After the adjustment for confounding factors, Spearman’s correlation analysis revealed that the GNRI was positively correlated with Ca, 25(OH)D, and PTH and negatively correlated with alkaline phosphatase (ALP) and procollagen of type-1 N-propeptide (P1NP). Regression analysis exhibited that the GNRI was significantly associated with osteoporosis.

The ROC curve analysis was performed using the GNRI as the test variable and the presence of osteoporosis as the status variable. This analysis yielded an AUC for the GNRI of 0.695 and was statistically significant (*P* < 0.05).

**Conclusions:**

A lower GNRI among T2DM patients in northern China is associated with a higher prevalence of osteoporosis.

**Supplementary Information:**

The online version contains supplementary material available at 10.1186/s12902-022-01215-z.

## Introduction

Osteoporosis is a highly prevalent disease among older adults. As age increases, osteoporosis and the increased risk of falls can lead to fractures, which can severely and negatively affect people’s quality of life and significantly increase the risks of hospitalization and death [1]. The incidence of diabetes is increasing as people’s dietary patterns are shifting toward higher energy levels. Hyperglycemia increases the production of advanced glycation end products and negatively affects bone mineralization, bone remodeling, and bone strength [2]. Diabetic complications can also considerably increase the risk of osteoporosis [3]. The prevalence of diabetic osteoporosis accounts for approximately > 50% of diabetic patients [4]. Moreover, the adverse outcomes of fracture are more severe in diabetic patients than in normoglycemic patients, and therefore, early identification of high-risk groups among elderly T2DM patients is critical [5].

Age, gender, vitamin D, muscle strength, and nutritional status are risk factors for osteoporosis. Elderly people are prone to malnutrition because of their specific metabolic characteristics and disease. Considerable evidence has proven that malnutrition is an independent risk factor for elderly patients with osteoporosis; studies have reported that low body weight, reduced ABL, and prealbumin can lead to an increased incidence of osteoporotic fractures [6, 7]. The geriatric nutritional risk index (GNRI) is used to assess the nutritional condition of older adults. It is calculated on the basis of serum ABL levels and the ratio of current actual body weight to ideal body weight [8].The GNRI allows for the early detection and diagnosis of malnutrition, timely and appropriate administration of interventions, as well as identification of conditions at risk for adverse effects, including cancer prognosis, postoperative complications, and mortality in patients on dialysis and those with cardiovascular disease [9–13]. Moreover, this tool is highly accurate and can be easily used clinically.

To our knowledge, few studies have evaluated the correlation between GNRI and osteoporosis. Bijuan Qing et al. [14] reported that the GNRI is associated with osteoporosis in geriatric patients. Liang Wang et al. [15] revealed that the GNRI is associated with osteoporosis as well as BMD in T2DM patients. Their study population comprised patients from southern China who had adequate vitamin D levels. However, in northern China, the majority of the population has vitamin D insufficiency or deficiency, is of an older age, and has a longer duration of diabetes; therefore, their study is not completely representative of the population with T2DM in China. Hence, the present study investigated the association between the GNRI and the presence of osteoporosis and assessed the value of GNRI for predicting osteoporosis in patients with T2DM.

## Materials and methods

### Methods

This was a cross-sectional observational study on 610 patients with T2DM of age ≥ 60 years who were treated at the Hebei General Hospital from January 2018 to December 2020. The diagnostic criteria of T2DM were based on the 1999 WHO. The study exclusion criteria for the participants were as follows: (1) individuals with diseases affecting the bone metabolism or the nutritional status, such as malignancies, severe liver diseases, kidney diseases, pituitary-related diseases, thyroid and parathyroid diseases, adrenal diseases, rheumatoid arthritis, and acute inflammatory diseases; (2) individuals who have been bedridden for a long period; (3) individuals who are taking drugs that affect their bone metabolisms, such as vitamin D, calcium, bisphosphonates, and glucocorticoids. This study protocol was approved by the Ethics Committee of the Hebei General Hospital and complies with the Declaration of Helsinki.

### Clinical information

Patient demographics and clinical characteristics, including information on gender, age, disease duration, and comorbidities, were collected from their respective medical records.

The weights of the patients were measured while they were wearing light clothing and their heights were measured without shoes. The body weight obtained was divided by height squared (kg/m^2^) to calculate the body mass index for each patient.

### Biochemical indicators

Serum samples were collected after fasting for at least 8 h. Triglycerides, total cholesterol, high-density lipoprotein cholesterol, low-density lipoprotein cholesterol, and albumin levels, fasting blood glucose, glycosylated hemoglobin, bone metabolism indicators, ALP, bone glaprotein (BGP), β-CTX, P1NP, 25(OH)D, and PTH were measured. In addition, other biochemical markers, such as uric acid, blood creatinine, and calcium were also analyzed.

### Bone mineral density

Bone densitometry was performed using a dual-energy X-ray bone densitometer to measure the bone density values in the lumbar spine (L1–4), femoral neck, and total hip. In accordance with the criteria for the definition of osteoporosis in 1994 WHO, T values ≤ -2.5 standard deviations were obtained for any part of the lumbar spine, femoral neck, or total hip [16].

### Calculating GNRI

The GNRI was calculated using the following formula:

- GNRI = [1.489 × albumin (g/dL)] + [41.7 × (body weight/WL0)].

WL0 represented the ideal body weight (kg), which was calculated as follows:

- For men: height (cm)-100-[(height (cm)-150)/4].
- For women: height (cm)-100-[(height (cm)-150)/2.5].
- Actual weight divided by the ideal weight was set to 1 where the actual weight exceeded the ideal weight.

According to the results obtained, there are 4 levels: GNRI < 82 indicating a high nutritional risk, GNRI:82 to < 92 indicating moderate nutritional risk, GNRI:92 to ≤ 98 indicating low risk, and GNRI > 98 indicating no risk.

### Statistical analysis

All data were statistically analyzed using the SPSS 25 software. Data distribution was evaluated with the Kolmogorov–Smirnov test. The mean ± standard deviation was applied to indicate that the data were subject to a normal distribution, while analysis of variance (ANOVA) was applied for comparisons between groups. The median (25th percentile, 75th percentile) was applied to indicate that the data did not conform to a normal distribution, and Mann–Whitney U-test was applied for comparisons between the groups. Categorical data were expressed as frequencies (%), and differences between the groups were determined using the χ2 test. Spearman correlational analysis was applied to determine the correlation among GNRI, BMD, and bone metabolism indicators. Logistic regression analysis was performed to assess the relationship between GNRI and osteoporosis. ROC curves were applied to assess the predictive properties of GNRI for osteoporosis and to calculate the area under the receiver operating characteristic curve (AUC).

## Results

### Clinical characteristics of the enrolled patients

**Fig. 1:**
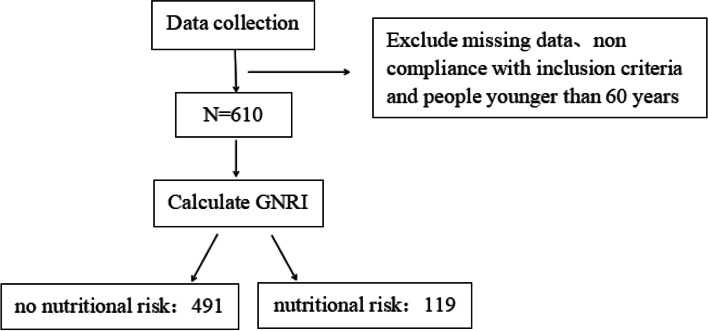
Flow chart depicting the subjects’ selection process

As shown in Fig. 1. Selection of Subjects，all patients were grouped according to the GNRI score and categorized into the no-nutritional risk group (GNRI > 98) and the nutritional risk group (GNRI ≤ 98) [17].

As shown in Table 1, when compared with the no-nutritional risk group, the nutritional risk group had a longer diabetes course, older age, lower BMI, lower ABL, higher HbA1c, lower TG, lower Ca, lower 25 (OH) D, lower PTH, lower femoral neck BMD, lower total hip BMD and higher prevalence of osteoporosis. The differences between the two groups were statistically significant (*P* < 0.05).

**Table 1:** Clinical characteristics of patients as stratified by their GNRI scores

Variables	Total	GNRI > 98	GNRI ≤ 98	*P*
	(*n* = 610)	(*n* = 491)	(*n* = 119)	
Age (years)	66.00(63.00,70.00)	66.00(63.00,69.00)	68.00(64.00,76.00)	0.000
Diabetes course (years)	10.00(5.00,17.00)	10.00(5.00,16.00)	15.00(6.00,20.00)	0.001
BMI (kg/m^2^)	26.07 ± 3.55	26.33 ± 3.43	24.99 ± 3.83	0.002
ALB (g/L)	41.10 ± 3.59	42.36 ± 2.57	35.93 ± 2.36	0.000
HbA1c (mmol/L)	8.12(6.95,9.50)	8.10(6.90,9.50)	9.20(7.40,10.80)	0.000
FPG (mmol/L)	7.01(5.77,9.39)	6.97(5.64,8.88)	7.45(5.93,10.03)	0.062
TC (mmol/L)	4.23(3.27,5.24)	4.25(3.25,5.33)	4.14(3.45,4.93)	0.336
TG (mmol/L)	1.23 (0.98,1.69)	1.24 (1.00,1.75)	1.12 (0.80,1.52)	0.001
HDL-c (mmol/L)	1.18(0.95,1.45)	1.19(0.96,1.48)	1.13(0.90,1.40)	0.065
LDL-c (mmol/L)	2.72(1.82,3.44)	2.76(1.78,3.51)	2.57(1.94,3.25)	0.286
Cr (umol/L)	73.30(61.48,98.25)	73.50(62.10,100.60)	71.00(60.00,94.10)	0.244
Uric (mmol/L)	277.45(203.90,340.68)	280.10(204.40,343.80)	255.00(197.80,330.20)	0.114
Ca (mmol/L)	2.30 (2.22,2.37)	2.33 (2.23,2.37)	2.26(2.18,2.34)	0.000
25(OH)D (ng/mL)	17.46(13.59,22.66)	18.18(14.24,23.37)	14.94(11.73,19.87)	0.000
ALP (IU/L)	72.25(51.18,89.85)	71.60(49.50,88.70)	73.80(55.80,93.50)	0.337
BGP (ng/mL)	12.75(9.87,16.48)	12.68(9.82,16.32)	13.16(10.01,16.55)	0.981
β-CTX (ng/mL)	0.35(0.24,0.51)	0.35(0.24,0.51)	0.36(0.25,0.51)	0.604
P1NP (ng/mL)	40.34(30.11,52.14)	39.65(29.89,51.86)	42.11(31.77,57.36)	0.132
PTH (ng/mL)	37.10(27.86,47.34)	37.82(28.45,48.02)	33.76(23.84,44.41)	0.004
BMD				
Total lumbar (g/cm^2^)	0.89 ± 0.16	0.89 ± 0.16	0.85 ± 0.16	0.138
Femur neck (g/cm^2^)	0.83 ± 0.16	0.83 ± 0.16	0.78 ± 0.13	0.017
Total hip (g/cm^2^)	0.70 ± 0.15	0.70 ± 0.15	0.65 ± 0.11	0.038
Osteoporosis%	25.9%	19.8%	51.3%	0.000

Annotation: *BMI* Body mass index, *ALB* Albumin, *HbA1c* Glycosylated hemoglobin, *FPG* Fasting plasma glucose, *TC* Total cholesterol, *TG* Triglyceride, *HDL-c* High-density

lipoprotein cholesterol, *LDL-c* Low-density lipoprotein cholesterol, *Cr* Creatinine, *Ca* Calcium, *25(OH)D* 25-hydroxy-vitamin-D, *ALP* Alkaline- phosphatase, *BGP* Bone glaprotein, *β-CTX* β-isomerized C-terminal telopeptides, *P1NP* Procollagen of type-1 N-propeptide, *PTH* Parathyroid hormone, *BMD* Bone mineral density

**Fig. 2:**
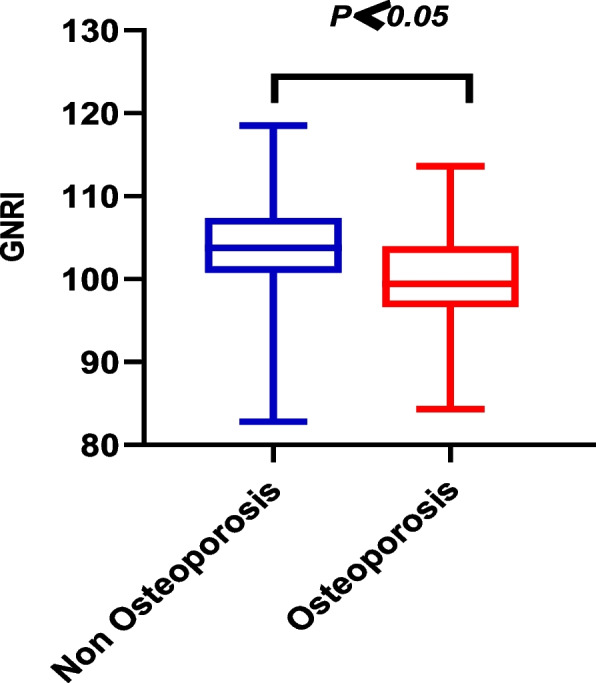
The chart depicting the GNRI score of the non-osteoporotic and osteoporotic groups

The patients were grouped according to the presence and absence of osteoporosis. Patients in the non-osteoporotic group had higher GNRI values when compared to those in the osteoporotic group (*P* < 0.05; Fig. 2).

### Spearman's correlations between the geriatric nutritional risk index and bone metabolism indicators

Spearman's correlation analysis yielded a positive correlation between GNRI and Ca, 25(OH)D, PTH. A negative correlation was recorded between GNRI and PINP. After adjusting for confounding factors, including age, the duration of diabetes, HbA1c, TC, TG, HDL, LDL, UA, Cr, and 24 h microalbuminuria (24 h-mAlb), GNRI was positively correlated with Ca, 25(OH)D, and PTH and negatively correlated with ALP and PINP (Table 2).

**Table 2:** Correlation between the Geriatric Nutrition Risk Index and the indicators of bone metabolism

Variables	Before adjusting		After adjusting	
	r	*P*	r	*P*
Ca (mmol/L)	0.173	0.000	0.107	0.009
25(OH)D (ng/mL)	0.269	0.000	0.208	0.000
ALP (IU/L)	0.035	0.392	-0.081	0.047
BGP (ng/mL)	0.012	0.769	-0.069	0.091
β-CTX (ng/mL)	-0.042	0.300	-0.057	0.164
P1NP (ng/mL)	-0.085	0.035	-0.132	0.001
PTH (ng/mL)	0.127	0.002	0.126	0.002

Annotation: After adjusting for confounding factors, including age, the duration of diabetes, HbA1c, TC, TG, UA, Cr, and 24 h-mAlb

**Fig. 3:**
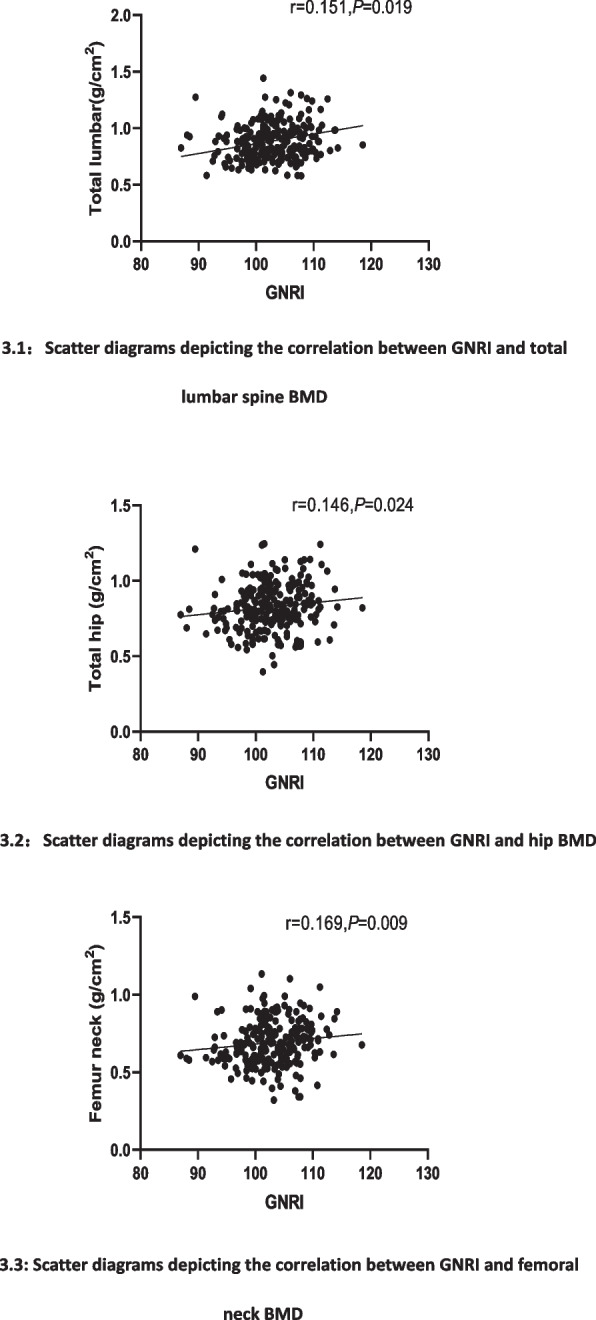
Scatter diagrams depicting the correlation between GNRI and BMD

Correlation analysis revealed that GNRI was positively correlated with lumbar BMD, femoral neck BMD, and total hip BMD (*P* < 0.05; Fig. 3).

### Logistic regression analysis of participants with osteoporosis

The results of the univariate logistic analysis suggest that GNRI, gender, age, duration of diabetes, 24 h-mAlb, 25(OH)D, P1NP, and FPG were associated with osteoporosis (Table 3).

**Table 3:** Univariate Logistic regression analysis of osteoporosis

Variables	SE	Odds ratio (95% CI)	*P*
Gender (male)	0.196	3.006 (.048, 4.412)	0.000
Age (years)	0.015	1.110 (1.069, 1.132)	0.000
Diabetes course (years)	0.012	1.037 (1.014, 1.061)	0.002
UA (mmol/L)	0.001	0.999(0.997, 1.000)	0.083
TC (mmol/L)	0.052	0.922 (0.832, 1.022)	0.124
TG (mmol/L)	0.073	0.953 (0.827, 1.100)	0.512
24 h-mAlb (mgL/24 h)	0.004	0.091 (0.984, 0.999)	0.002
Cr (umol/L)	0.001	1.000(0.999, 1.002)	0.668
Ca (mmol/L)	0.714	0.403 (0.099, 1.632)	0.203
25 (OH) D (ng/mL)	0.013	0.969 (0.944, 0.994)	0.015
ALP (IU/L)	0.002	0.998 (0.994, 1.003)	0.457
P1NP (ng/mL)	0.004	1.011 (1.004, 1.019)	0.003
PTH (ng/mL)	0.005	1.010(1.000, 1.020)	0.060
HbA1c (mmol/L)	0.050	0.980 (0.889, 1.080)	0.679
FPG (mmol/L)	0.027	1.069 (1.014, 1.127)	0.014
GNRI	0.018	0.885(0.854, 0.917)	0.000

Multivariate analysis of the association between GNRI and osteoporosis through logistic regression (Table 4). After adjusting for gender, age, the duration of diabetes, FPG, 25(OH)D, P1NP, and 24 h-mAlb a significant association was established between GNRI and osteoporosis.

**Table 4:** Multivariate logistic regression analysis of osteoporosis

	SE	OR (95% CI)	*P*
GNRI	0.020	0.908 (0.873, 0.945)	0.000

Annotation: Gender, age, diabetes course, FPG, 25 (OH)D, P1NP, and 24 h-mAlb are involved in the logistic multivariate regression analysis. *SE* Standard error

Using GNRI as the categorical variable, the risk of osteoporosis in the nutritional risk group was 3.31-times higher than that in the non-nutritional risk group (Table 5).

**Table 5:** Multivariate logistic regression analysis of osteoporosis

	SE	OR (95% CI)	*P*
GNRI (GNRI < 98)	0.238	3.331 (2.077, 5.275)	0.000

Annotation: Gender, age, diabetes course, FPG, 25 (OH)D, P1NP, and 24 h-mAlb are involved in the logistic multivariate regression analysis. *SE S*tandard error

### Predictive properties of GNRI for osteoporosis

**Fig. 4:**
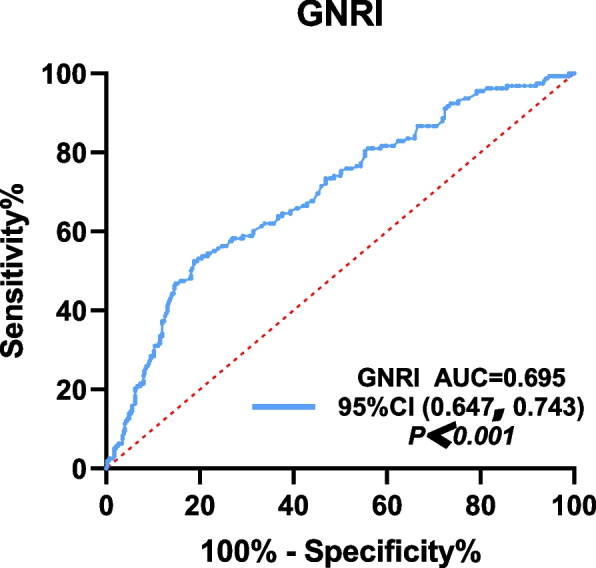
Receiver operating characteristic curve of osteoporosis

ROC curve analysis was performed with GNRI as the test variable and the presence of osteoporosis as the status variable (Fig. 4). The analysis yielded an area under the curve for GNRI of 0.695, 95% confidence intervals of (0.647,0.743), with an optimal GNRI threshold of 99.56 for predicting osteoporosis, and a sensitivity of 81.19% and specificity of 52.53%.

## Discussion

The GNRI is an indicator of nutritional status in elderly people. It is calculated using serum ABL level, weight, and height. Moreover, it involves a dual assessment of serum ABL and BMI that complements and improves its diagnostic accuracy. Good nutritional status plays a good role in bone metabolism. Similarly, malnutrition increases the risk of osteoporosis and fragility fractures [8].

Therefore, awareness is required to prevent complications associated with malnutrition in elderly people with high fracture risk. Several recent studies on GNRI and osteoporosis have reported a significant correlation between the two. Tokumoto et al. reported that a lower GNRI value is a risk factor for femoral neck BMD in patients receiving modified biological disease antirheumatic drugs for rheumatoid arthritis (RA) [18]. A positive correlation was observed between the GNRI and BMD as well as the T-score in patients undergoing hemodialysis and total thyroidectomy [19, 20]. In a study[21], 57% of 858 women with with hip fractures were at a high or moderate risk of nutrition. Overall, the GNRI has the potential to early identify patients at a high risk of osteoporosis among the elderly T2DM population.

In the present study, we investigated the correlation between GNRI and osteoporosis in T2DM patients from northern China. Our results yield that low levels of GNRI have a negative effect on bone metabolism in patients. According to the correlation analysis, the GNRI was positively correlated with BMD (*P* < 0.05). The logistic regression analysis revealed a significant association of the GNRI with osteoporosis. The ROC analysis yielded a GNRI cut-off value of 99.56. Our results providing a theoretical basis for screening for osteoporosis clinically.

The current understanding of the possible mechanism underlying the association between the GNRI and osteoporosis is as follows. First, malnutrition affects calcium and vitamin D intake, which may increase bone mineral loss in patients, making bone mineralization difficult and leading to osteoporosis. Second, hypoalbuminemia is a marker of both nutritional status and chronic inflammatory response. Hypoalbuminemia activates osteoclasts and inhibits osteoblasts through NF-κB factors and other inflammatory cytokines[22]. Moreover, hypoalbuminemia causes a decrease in insulin-like growth factor-1 synthesis, thereby leading to a decreased number of osteoblasts, decreased cellular activity, increased osteoclast lifespan, increased bone resorption, and decreased bone remodeling [23]. Finally, hypoproteinemia is associated with inadequate muscle synthesis and decreased skeletal muscle mass, resulting in decreased balance and gait capacity, which is associated with the risk of falls and fractures [24, 25].

Liang Wang et al. found no correlation between GNRI and 25(OH)D. By contrast, we yielded a positive correlation between GNRI and 25(OH)D. A possible reason for our different results is that in southern China even though nutritional status indicates reduced dietary vitamin intake, vitamin D levels can still be ensured with adequate sun exposure. By contrast, in northern China, insufficient sun exposure and nutritional barriers make people more prone to vitamin D insufficiency and deficiency. Studies have shown a close relationship between latitude sunlight deficiency, skin coverage, and vitamin D. In China, a clear geographical division of vitamin D deficiency can be seen, with populations in northern, northeastern, and northwestern China at the north of 35 degrees north latitude being more severely undernourished, whereas vitamin D levels are adequate in areas south of 25 degrees north latitude and the middle of the country [26, 27].

In our study, GNRI was negatively correlated with P1NP. A possible reason for this negative correlation is that PINP secreted by osteoblasts is a known marker of bone formation and reflects collagen formation and osteoblast activation. During bone turnover, bone formation and resorption are tightly coupled, with accelerated bone turnover predisposing a person to bone loss. Therefore, high GNRI values are associated with low bone turnover and reduced bone loss. GNRI was positiveiy correlated with PTH. A possible reason is that a positive correlation was observed between serum PTH and BMI and fat mass. Mehrotra indicated that reduced PTH is a risk factor for malnutrition [28, 29]. PTH promotes the inward flow of calcium ions into adipocytes and stimulates adipose synthesis. Accordingly, low PTH levels inhibit the adipose synthesis and cause protein depletion. Bone-specific alkaline phosphatase (BALP) is closely related to normal bone growth and development. This enzyme is a marker of osteoblast maturation and activation. However, the specificity of our current assay is not good. Moreover, BALP has some crossover with liver-derived ALP, and we measured total ALP and not BALP. Therefore, the relationship of the GNRI with ALP does not accurately reflect the bone metabolism level.

Logistic regression analysis revealed that advanced age is an independent risk factor for osteoporosis, with bone density decreasing every year with an increase in age. Furthermore, oxidative stress, which increases osteoclast activity and bone resorption, is a cause of age-related bone loss. According to the present study, diabetes duration is an influential factor for osteoporosis in T2DM patients. The relative risk of diabetes course increases from 1.40 (1. 08–1. 82) at less than 5 years to 2.66 (2. 04–3. 47) at greater than 15 years [30]. The non-enzymatic glycosylation response of T2DM contributes to the decline in bone mass. Because of hyperglycemia, advanced glycosylation end products are accumulated in the organic bone matrix, thereby resulting in stiffening of type I collagen in the bone matrix, decreased bone strength, increased bone fragility, and promotion of osteoblast apoptosis [31]. Our results suggest that suboptimal glycemic control is also an independent risk factor for osteoporosis development.

Furthermore, based on our regression analysis of the GNRI and osteoporosis, we concluded that uric acid (UA) is not associated with osteoporosis and is neither a protective nor a risk factor for osteoporosis. Several studies have reported that higher UA levels are protective against osteoporosis [32–34]. Our results were different from those of these studies may be because of differences in gender, region, ethnicity, study methodology, and sample size. Finally, the association of serum UA with osteoporosis may be directly or indirectly confounded by the fact that many older adults have two or more chronic diseases, such as obesity and DM.

This study has some limitations. First, this cross-sectional study does not offer a mechanism-related explanation for the observed association, This study also does not indicate a causal relationship between the GNRI and osteoporosis. Second, the serum data of the T2DM patients and BMD were both collected only once, thus leading to bias. Third, some relevant parameters affecting the study results may have been overlooked, such as a history of smoking and alcohol consumption, hormone levels, dietary habits, exercise situation, and history of previous fractures.

In summary, our results demonstrate that a lower GNRI is associated with a higher prevalence of osteoporosis and that the GNRI is an easy-to-use tool for assessing nutritional status and osteoporosis in T2DM patients. Nutritional supplementation therapy may reduce osteoporosis prevalence in T2DM patients.

## Conclusions

A lower GNRI among T2DM patients in northern China is associated with a higher prevalence of osteoporosis.

## Supplementary Information

**Additional file 1**: **Figure 1.** Flow chart depicting the subjects’ selection process.

**Additional file 2**: **Table 1.** Clinical characteristics of patients as stratified by their GNRI scores.

**Additional file 3**: **Figure 2.** The chart depicting the GNRI score of the non-osteoporotic and osteoporotic groups.

**Additional file 4**: **Table 2.** Correlation between the Geriatric Nutrition Risk Index and the indicators of bone metabolism.

**Additional file 5**: **Figures 3.1.** Scatter diagrams depicting the correlation between GNRI and total lumbar spine BMD. **Figure 3.2. **Scatter diagrams depicting the correlation between GNRI and hip BMD. **Figure 3.3.** Scatter diagrams depicting the correlation between GNRI and femoral neck BMD.

**Additional file 6**: **Table 3.** Univariate Logistic regression analysis of osteoporosis.

**Additional file 7**: **Table 4.** Multivariate logistic regression analysis of osteoporosis.

**Additional file 8**: **Table 5.** Multivariate logistic regression analysis of osteoporosis.

**Additional file 9**: **Figure 4.** Receiver operating characteristic curve of osteoporosis.

**Additional file 10**: Abbreviations T2DM: Type 2 diabetes mellitus. OP: Osteoporosis. BMD: Bone mineral density. GNRI: Geriatric Nutrition Risk Index. ALB: Albumin. BMI: Body mass index. HbA1c: Glycosylated hemoglobin. FPG: Fasting plasma glucose. TC: Total cholesterol. TG: Triglyceride. HDL-c: High-density lipoprotein cholesterol. LDL-c: Low-density lipoprotein cholesterol. UA: Uric Acid. Cr: Creatinine. Ca: Calcium. ALP: Alkaline phosphatase. 25（OH）D: 25-hydroxy-vitamin. BGP: Bone glaprotein. β-CTX: β-isomerized C-terminal telopeptides. P1NP: Procollagen of type 1 N-propeptide. PTH: Parathyroid hormone. ROC: Receiver Operating Characteristic Curve. AUC: Area under the cure.

## Data Availability

The datasets supporting the conclusions of this article is included within the article.

## Abbreviations

T2DM: Type 2 diabetes mellitus
OP: Osteoporosis
BMD: Bone mineral density
GNRI: Geriatric Nutrition Risk Index
ALB: Albumin
BMI: Body mass index
HbA1c: Glycosylated hemoglobin
FPG: Fasting plasma glucose
TC: Total cholesterol
TG: Triglyceride
HDL-c: High-density lipoprotein cholesterol
LDL-c: Low-density lipoprotein cholesterol
UA: Uric Acid
Cr: Creatinine
Ca: Calcium
ALP: Alkaline phosphatase
25(OH)D: 25-Hydroxy-vitamin-D
BGP: Bone glaprotein
β-CTX: β-isomerized C-terminal telopeptides
P1NP: Procollagen of type 1 N-propeptide
PTH: Parathyroid hormone
ROC: Receiver Operating Characteristic Curve
AUC: Area under the cure

## References

[1] Cianferotti L, Bertoldo F, Bischoff-Ferrari HA (2017). Vitamin D supplementation in the prevention and management of major chronic diseases not related to mineral homeostasis in adults: research for evidence and a scientific statement from the European society for clinical and economic aspects of osteoporosis and osteoarthritis (ESCEO)[J]. Endocrine.

[2] Dede AD, Tournis S, Dontas I (2014). Type 2 diabetes mellitus and fracture risk[J]. Metab.

[3] Vestergaard P, Rejnmark L, Mosekilde L (2009). Diabetes and its complications and their relationship with risk of fractures in type 1 and 2 diabetes[J]. Calcif Tissue Int.

[4] Kumeda Y, Inaba M (2002). Diabetic osteoporosis][J. Nihon Rinsho.

[5] Sellmeyer DE, Civitelli R, Hofbauer LC (2016). Skeletal Metabolism, Fracture Risk, and Fracture Outcomes in Type 1 and Type 2 Diabetes[J]. Diabetes.

[6] Xiu S, Chhetri JK, Sun L (2019). Association of serum prealbumin with risk of osteoporosis in older adults with type 2 diabetes mellitus: a cross-sectional study[J]. Ther Adv Chronic Dis.

[7] Coin A, Sergi G, Beninca P (2000). Bone mineral density and body composition in underweight and normal elderly subjects[J]. Osteoporos Int.

[8] Bouillanne O, Morineau G, Dupont C (2005). Geriatric Nutritional Risk Index: a new index for evaluating at-risk elderly medical patients[J]. Am J Clin Nutr.

[9] Tang M, Li L, Zhang P, et al. The Geriatric Nutritional Risk Index Predicts Overall Survival in Geriatric Patients with Metastatic Lung Adenocarcinoma[J]. Nutrition and cancer. 2020:1–9. 10.1080/01635581.2020.1743865 32238011

[10] Xu J, Zhou X, Zheng C (2019). The geriatric nutritional risk index independently predicts adverse outcomes in patients with pyogenic liver abscess[J]. BMC Geriatr.

[11] Funamizu N, Omura K, Takada Y, et al. Geriatric Nutritional Risk Index Less Than 92 Is a Predictor for Late Postpancreatectomy Hemorrhage Following Pancreatoduodenectomy: A Retrospective Cohort Study[J]. Cancers (Basel), 2020,12(10). 10.3390/cancers12102779 PMC7600944 32998260

[12] Sasaki M, Miyoshi N, Fujino S (2020). The Geriatric Nutritional Risk Index predicts postoperative complications and prognosis in elderly patients with colorectal cancer after curative surgery[J]. Sci Rep.

[13] Funamizu N, Omura K, Ozaki T (2020). Geriatric nutritional risk index serves as risk factor of surgical site infection after pancreatoduodenectomy: a validation cohort Ageo study[J]. Gland Surg.

[14] Qing B, Wang N, Wang L (2021). Association between geriatric nutrition risk index and bone mineral density in elderly Chinese people[J]. Arch Osteoporos.

[15] Wang L, Zhang D, Xu J (2020). Association between the Geriatric Nutritional Risk Index, bone mineral density and osteoporosis in type 2 diabetes patients[J]. J Diabetes Investigation.

[16] Assessment of fracture risk and its application to screening for post-menopausal osteoporosis. Report of a WHO Study Group. World Health Organ Tech Rep Series. 1994;843:1–129. 7941614

[17] Sakamoto T, Yagyu T, Uchinaka E (2021). The prognostic significance of combined geriatric nutritional risk index and psoas muscle volume in older patients with pancreatic cancer[J]. BMC Cancer.

[18] Tokumoto H, Tominaga H, Arishima Y (2018). Association between Bone Mineral Density of Femoral Neck and Geriatric Nutritional Risk Index in Rheumatoid Arthritis Patients Treated with Biological Disease-Modifying Anti-Rheumatic Drugs[J]. Nutrients.

[19] Chen S C, Chung W S, Wu P Y, et al. Associations among Geriatric Nutrition Risk Index, bone mineral density, body composition and handgrip strength in patients receiving hemodialysis[J]. Nutrition, 2019,65:6-12. 10.1016/j.nut.2019.02.013 31029923

[20] Chiu TH, Chen SC, Yu HC (2020). Association between Geriatric Nutrition Risk Index and Skeletal Muscle Mass Index with Bone Mineral Density in Post-Menopausal Women Who Have Undergone Total Thyroidectomy[J]. Nutrients.

[21] Di Monaco M, Castiglioni C, Bardesono F (2020). Simultaneous hip and upper-limb fractures are associated with lower Geriatric Nutritional Index scores than isolated hip fractures: a cross-sectional study of 858 women[J]. Aging Clin Exp Res.

[22] Zheng CM, Wu CC, Lu CL (2019). Hypoalbuminemia differently affects the serum bone turnover markers in hemodialysis patients[J]. Int J Med Sci.

[23] Afshinnia F, Wong KK, Sundaram B (2016). Hypoalbuminemia and Osteoporosis: Reappraisal of a Controversy[J]. J Clin Endocrinol Metab.

[24] Al-Jebawi AF, YoussefAgha AH, Al SH (2017). Attenuated PTH responsiveness to vitamin D deficiency among patients with type 2 diabetes and chronic hyperglycemia[J]. Diabetes Res Clin Pract.

[25] Sotunde OF, Kruger HS, Wright HH (2015). Lean Mass Appears to Be More Strongly Associated with Bone Health than Fat Mass in Urban Black South African Women[J]. J Nutr Health Aging.

[26] Gennari C (2001). Calcium and vitamin D nutrition and bone disease of the elderly[J]. Public Health Nutr.

[27] Man PW, van der Meer IM, Lips P (2016). Vitamin D status and bone mineral density in the Chinese population: a review[J]. Arch Osteoporos.

[28] Kamycheva E, Sundsfjord J, Jorde R (2004). Serum parathyroid hormone level is associated with body mass index. The 5th Tromso study[J]. Eur J Endocrinol.

[29] Mehrotra R, Supasyndh O, Berman N (2004). Age-related decline in serum parathyroid hormone in maintenance hemodialysis patients is independent of inflammation and dietary nutrient intake[J]. J Ren Nutr.

[30] Koh WP, Wang R, Ang LW (2010). Diabetes and risk of hip fracture in the Singapore Chinese Health Study[J]. Diabetes Care.

[31] Alikhani M, Alikhani Z, Boyd C (2007). Advanced glycation end products stimulate osteoblast apoptosis via the MAP kinase and cytosolic apoptotic pathways[J]. Bone.

[32] Ahn SH, Lee SH, Kim BJ (2013). Higher serum uric acid is associated with higher bone mass, lower bone turnover, and lower prevalence of vertebral fracture in healthy postmenopausal women[J]. Osteoporos Int.

[33] De Pergola G, Giagulli VA, Bartolomeo N (2017). Independent Relationship between Serum Osteocalcin and Uric Acid in a Cohort of Apparently Healthy Obese Subjects[J]. Endocr Metab Immune Disord Drug Targets.

[34] Makovey J, Macara M, Chen JS (2013). Serum uric acid plays a protective role for bone loss in peri- and postmenopausal women: a longitudinal study[J]. Bone.

