# Supplementary figures and images for: Relationship between geriatric nutritional risk index and osteoporosis in type 2 diabetes in Northern China
Source: BMC Endocr Disord. 2022 Dec 9;22:308. doi: 10.1186/s12902-022-01215-z (PMC9733244; doi:10.1186/s12902-022-01215-z)

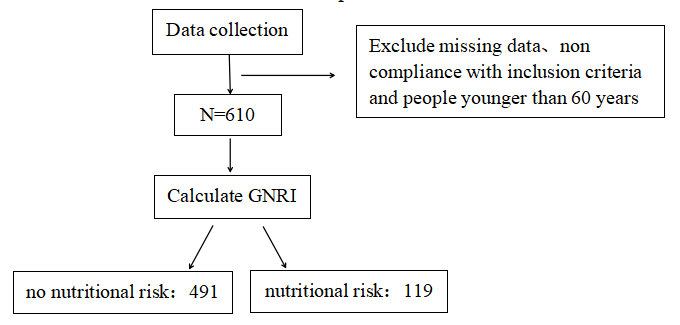


**Figure 1 Flow chart depicting the subjects’ selection process**

Supplement: Supplementary file 1 — Additional file 1: Figure 1. Flow chart depicting the subjects’ selection process. [file 12902_2022_1215_MOESM1_ESM.docx]
