# Supplementary material for: Relationship between geriatric nutritional risk index and osteoporosis in type 2 diabetes in Northern China
Source: BMC Endocr Disord. 2022 Dec 9;22:308. doi: 10.1186/s12902-022-01215-z (PMC9733244; doi:10.1186/s12902-022-01215-z)
Supplement: Supplementary file 2 — Additional file 2: Table 1. Clinical characteristics of patients as stratified by their GNRI scores. [file 12902_2022_1215_MOESM2_ESM.docx]

**Table 1 Clinical characteristics of patients as stratified by their GNRI scores**

| Variables | Total | GNRI ＞98 | GNRI ≤98 | *P* |
| --- | --- | --- | --- | --- |
|  | (n = 610) | (n = 491) | (n = 119) |  |
| Age (years) | 66.00(63.00,70.00) | 66.00(63.00,69.00) | 68.00(64.00,76.00) | 0.000 |
| Diabetes duration (years) | 10.00(5.00,17.00) | 10.00(5.00,16.00) | 15.00(6.00,20.00) | 0.001 |
| BMI (kg/m^2^) | 26.07 ±3.55 | 26.33 ±3.43 | 24.99 ±3.83 | 0.002 |
| ALB (g/L) | 41.10 ±3.59 | 42.36 ±2.57 | 35.93 ±2.36 | 0.000 |
| HbA1c (mmol/L) | 8.12(6.95,9.50) | 8.10(6.90,9.50) | 9.20(7.40,10.80) | 0.000 |
| FPG (mmol/L) | 7.01(5.77,9.39) | 6.97(5.64,8.88) | 7.45(5.93,10.03) | 0.062 |
| TC (mmol/L) | 4.23(3.27,5.24) | 4.25(3.25,5.33) | 4.14(3.45,4.93) | 0.336 |
| TG (mmol/L) | 1.23 (0.98,1.69) | 1.24 (1.00,1.75) | 1.12 (0.80,1.52) | 0.001 |
| HDL (mmol/L) | 1.18(0.95,1.45) | 1.19(0.96,1.48) | 1.13(0.90,1.40) | 0.065 |
| LDL (mmol/L) | 2.72(1.82,3.44) | 2.76(1.78,3.51) | 2.57(1.94,3.25) | 0.286 |
| Cr (umol/L) | 73.30(61.48,98.25) | 73.50(62.10,100.60) | 71.00(60.00,94.10) | 0.244 |
| Uric (mmol/L) | 277.45(203.90,340.68) | 280.10(204.40,343.80) | 255.00(197.80,330.20) | 0.114 |
| Ca (mmol/L) | 2.30 (2.22,2.37) | 2.33 (2.23,2.37) | 2.26(2.18,2.34) | 0.000 |
| 25(OH)D (ng/mL) | 17.46(13.59,22.66) | 18.18(14.24,23.37) | 14.94(11.73,19.87) | 0.000 |
| ALP (IU/L) | 72.25(51.18,89.85) | 71.60(49.50,88.70) | 73.80(55.80,93.50) | 0.337 |
| BGP (ng/mL) | 12.75(9.87,16.48) | 12.68(9.82,16.32) | 13.16(10.01,16.55) | 0.981 |
| β-CTX (ng/mL) | 0.35(0.24,0.51) | 0.35(0.24,0.51) | 0.36(0.25,0.51) | 0.604 |
| P1NP (ng/mL) | 40.34(30.11,52.14) | 39.65(29.89,51.86) | 42.11(31.77,57.36) | 0.132 |
| PTH (ng/mL) | 37.10(27.86,47.34) | 37.82(28.45,48.02) | 33.76(23.84,44.41) | 0.004 |
| BMD |  |  |  |  |
| Total lumbar (g/cm^2^) | 0.89 ±0.16 | 0.89 ±0.16 | 0.85 ±0.16 | 0.138 |
| Femur neck (g/cm^2^) | 0.83 ±0.16 | 0.83 ±0.16 | 0.78 ±0.13 | 0.017 |
| Total hip (g/cm^2^) | 0.70 ±0.15 | 0.70 ±0.15 | 0.65 ±0.11 | 0.038 |
| Osteoporosis% | 25.9% | 19.8% | 51.3% | 0.000 |

Annotation: BMI, body mass index; ALB, albumin; HbA1c,glycosylated hemoglobin;

FPG, fasting plasma glucose; TC, total cholesterol; TG, triglyceride; HDL, high-density

lipoprotein cholesterol; LDL, low-density lipoprotein cholesterol; Cr, creatinine;Calcium,Ca; 25(OH)D, 25-hydroxy-vitamin-D; ALP, alkaline- phosphatase; BGP, bone glaprotein;β-CTX, β-isomerized C-terminal telopeptides; P1NP, procollagen of type-I N-propeptide; PTH, parathyroid hormone; BMD, bone mineral density.
