# Supplementary material for: Relationship between geriatric nutritional risk index and osteoporosis in type 2 diabetes in Northern China
Source: BMC Endocr Disord. 2022 Dec 9;22:308. doi: 10.1186/s12902-022-01215-z (PMC9733244; doi:10.1186/s12902-022-01215-z)
Supplement: Supplementary file 3 — Additional file 3: Figure 2. The chart depicting the GNRI score of the non-osteoporotic and osteoporotic groups. [file 12902_2022_1215_MOESM3_ESM.docx]

**Figure 2 The chart depicting the GNRI score of the non-osteoporotic and osteoporotic groups.**
