# Supplementary material for: Relationship between geriatric nutritional risk index and osteoporosis in type 2 diabetes in Northern China
Source: BMC Endocr Disord. 2022 Dec 9;22:308. doi: 10.1186/s12902-022-01215-z (PMC9733244; doi:10.1186/s12902-022-01215-z)
Supplement: Supplementary file 4 — Additional file 4: Table 2. Correlation between the Geriatric Nutrition Risk Index and the indicators of bone metabolism. [file 12902_2022_1215_MOESM4_ESM.docx]

**Table 2 Correlation between the Geriatric Nutrition Risk Index and the indicators of bone metabolism**

| Variables | Before adjusting | |  | After adjusting | |
| --- | --- | --- | --- | --- | --- |
|  | r | *P* |  | r | *P* |
| Ca (mmol/L) | 0.173 | 0.000 |  | 0.107 | 0.009 |
| 25(OH)D (ng/mL) | 0.269 | 0.000 |  | 0.208 | 0.000 |
| ALP (IU/L) | 0.035 | 0.392 |  | -0.081 | 0.047 |
| BGP (ng/mL) | 0.012 | 0.769 |  | -0.069 | 0.091 |
| β-CTX (ng/mL) | -0.042 | 0.300 |  | -0.057 | 0.164 |
| PINP (ng/mL) | -0.085 | 0.035 |  | -0.132 | 0.001 |
| PTH (ng/mL) | 0.127 | 0.002 |  | 0.126 | 0.002 |

Annotation: After adjusting for confounding factors, including age, the duration of diabetes, HbA1c, TC, TG，UA, Cr, and 24h-mAlb.
