# Supplementary material for: Relationship between geriatric nutritional risk index and osteoporosis in type 2 diabetes in Northern China
Source: BMC Endocr Disord. 2022 Dec 9;22:308. doi: 10.1186/s12902-022-01215-z (PMC9733244; doi:10.1186/s12902-022-01215-z)
Supplement: Supplementary file 5 — Additional file 5: Figures 3.1. Scatter diagrams depicting the correlation between GNRI and total lumbar spine BMD. Figure 3.2. Scatter diagrams depicting the correlation between GNRI and hip BMD. Figure 3.3. Scatter diagrams depicting the correlation between GNRI and femoral neck BMD. [file 12902_2022_1215_MOESM5_ESM.docx]

**Figures 3.1：Scatter diagrams depicting the correlation between GNRI and total lumbar spine BMD**

**Figure 3.2：Scatter diagrams depicting the correlation between GNRI and hip BMD**

**Figure 3.3: Scatter diagrams depicting the correlation between GNRI and femoral neck BMD**
