# Supplementary material for: Relationship between geriatric nutritional risk index and osteoporosis in type 2 diabetes in Northern China
Source: BMC Endocr Disord. 2022 Dec 9;22:308. doi: 10.1186/s12902-022-01215-z (PMC9733244; doi:10.1186/s12902-022-01215-z)
Supplement: Supplementary file 6 — Additional file 6: Table 3. Univariate Logistic regression analysis of osteoporosis. [file 12902_2022_1215_MOESM6_ESM.docx]

**Table 3: Univariate Logistic regression analysis of osteoporosis**

| Variables | SE | Odds ratio (95% CI) | *P* |
| --- | --- | --- | --- |
| Gender (male) | 0.196 | 3.006 (.048, 4.412) | 0.000 |
| Age (years) | 0.015 | 1.110 (1.069, 1.132) | 0.000 |
| Diabetes duration | 0.012 | 1.037 (1.014, 1.061) | 0.002 |
| UA (mmol/L) | 0.001 | 0.999(0.997, 1.000) | 0.083 |
| TC (mmol/L) | 0.052 | 0.922 (0.832, 1.022) | 0.124 |
| TG (mmol/L) | 0.073 | 0.953 (0.827, 1.100) | 0.512 |
| 24h-mAlb (mgL/24 h) | 0.004 | 0.091 (0.984, 0.999) | 0.002 |
| Cr (umol/L) | 0.001 | 1.000(0.999, 1.002) | 0.668 |
| Ca (mmol/L) | 0.714 | 0.403 (0.099, 1.632) | 0.203 |
| 25 (OH) D (ng/mL) | 0.013 | 0.969 (0.944, 0.994) | 0.015 |
| ALP (IU/L) | 0.002 | 0.998 (0.994, 1.003) | 0.457 |
| P1NP (ng/mL) | 0.004 | 1.011 (1.004, 1.019) | 0.003 |
| PTH (ng/mL) | 0.005 | 1.010(1.000, 1.020) | 0.060 |
| HbA1c (mmol/L) | 0.050 | 0.980 (0.889, 1.080) | 0.679 |
| FPG (mmol/L) | 0.027 | 1.069 (1.014, 1.127) | 0.014 |
| GNRI | 0.018 | 0.885(0.854, 0.917) | 0.000 |
