# Supplementary material for: Relationship between geriatric nutritional risk index and osteoporosis in type 2 diabetes in Northern China
Source: BMC Endocr Disord. 2022 Dec 9;22:308. doi: 10.1186/s12902-022-01215-z (PMC9733244; doi:10.1186/s12902-022-01215-z)
Supplement: Supplementary file 7 — Additional file 7: Table 4. Multivariate logistic regression analysis of osteoporosis. [file 12902_2022_1215_MOESM7_ESM.docx]

**Table 4: Multivariate logistic regression analysis of osteoporosis**

|  | SE | OR (95% CI) | *P* |
| --- | --- | --- | --- |
| GNRI | 0.020 | 0.908 (0.873, 0.945) | 0.000 |

Annotation: Gender, age, diabetes duration, FPG, 25 (OH)D, P1NP, and 24h-mAlb are involved in the logistic multivariate regression analysis. SE, standard error.
