# Supplementary material for: Relationship between geriatric nutritional risk index and osteoporosis in type 2 diabetes in Northern China
Source: BMC Endocr Disord. 2022 Dec 9;22:308. doi: 10.1186/s12902-022-01215-z (PMC9733244; doi:10.1186/s12902-022-01215-z)
Supplement: Supplementary file 8 — Additional file 8: Table 5. Multivariate logistic regression analysis of osteoporosis. [file 12902_2022_1215_MOESM8_ESM.docx]

**Table 5: Multivariate logistic regression analysis of osteoporosis**

|  | SE | OR (95% CI) | *P* |
| --- | --- | --- | --- |
| GNRI (GNRI ＜98) | 0.238 | 3.331 (2.077, 5.275) | 0.000 |

Annotation: Gender, age, diabetes duration, FPG, 25 (OH)D, P1NP, and 24h-mAlb are involved in the logistic multivariate regression analysis. SE, standard error.
