# Supplementary material for: Relationship between geriatric nutritional risk index and osteoporosis in type 2 diabetes in Northern China
Source: BMC Endocr Disord. 2022 Dec 9;22:308. doi: 10.1186/s12902-022-01215-z (PMC9733244; doi:10.1186/s12902-022-01215-z)
Supplement: Supplementary file 10 — Additional file 10: Abbreviations T2DM: Type 2 diabetes mellitus. OP: Osteoporosis. BMD: Bone mineral density. GNRI: Geriatric Nutrition Risk Index. ALB: Albumin. BMI: Body mass index. HbA1c: Glycosylated hemoglobin. FPG: Fasting plasma glucose. TC: Total cholesterol. TG: Triglyceride. HDL-c: High-density lipoprotein cholesterol. LDL-c: Low-density lipoprotein cholesterol. UA: Uric Acid. Cr: Creatinine. Ca: Calcium. ALP: Alkaline phosphatase. 25（OH）D: 25-hydroxy-vitamin. BGP: Bone glaprotein. β-CTX: β-isomerized C-terminal telopeptides. P1NP: Procollagen of type 1 N-propeptide. PTH: Parathyroid hormone. ROC: Receiver Operating Characteristic Curve. AUC: Area under the cure. [file 12902_2022_1215_MOESM10_ESM.docx]

**Abbreviations**

| Acronym | Full Name |
| --- | --- |
| T2DM | Type 2 diabetes mellitus |
| OP | Osteoporosis |
| BMD | Bone mineral density |
| GNRI | Geriatric Nutrition Risk Index |
| ALB | Albumin |
| BMI | [Body](javascript:;) [mass](javascript:;) [index](javascript:;) |
| HbA1c | Glycosylated hemoglobin |
| FPG | Fasting plasma glucose |
| TC | Total cholesterol |
| TG | Triglyceride |
| HDL | [High-density](javascript:;) [lipoprotein](javascript:;) |
| LDL | [Low-density](javascript:;) [lipoprotein](javascript:;) |
| UA | [Uric](javascript:;) [Acid](javascript:;) |
| Cr | Creatinine |
| Ca | Calcium |
| ALP | Alkaline phosphatase |
| 25（OH）D | 25-hydroxy-vitamin |
| BGP | Bone glaprotein |
| β-CTX | β-isomerized C-terminal telopeptides |
| P1NP | Procollagen of type I N-propeptide |
| PTH | Parathyroid hormone |
| ROC | Receiver Operating Characteristic Curve |
| AUC | Area under the cure |
